# Transcriptomic analysis reveals mechanisms for the different drought tolerance of sweet potatoes

**DOI:** 10.3389/fpls.2023.1136709

**Published:** 2023-03-16

**Authors:** Enliang Liu, Linli Xu, Zhengqian Luo, Zhiqiang Li, Guohui Zhou, Haifeng Gao, Furong Fang, Jun Tang, Yue Zhao, Zhilin Zhou, Ping Jin

**Affiliations:** ^1^ Grain Crops Institute, Xinjiang Academy of Agricultural Sciences, Urumqi, China; ^2^ Comprehensive Proving Ground, Xinjiang Academy of Agricultural Sciences, Urumqi, China; ^3^ Adsen Biotechnology Co., Ltd., Urumqi, China; ^4^ Institute of Plant Protection, Xinjiang Academy of Agricultural Sciences, Urumqi, China; ^5^ Xuzhou Institute of Agricultural Sciences in Xuhuai District, Xuzhou, China

**Keywords:** sweet potato, drought stress, transcriptomics, differentially expressed genes, drought tolerance

## Abstract

Drought is a common environmental stress with great negative impacts on plant growth, development and geographical distribution as well as agriculture and food production. Sweet potato is characterized by starchy, fresh and pigmented tuber, and is regarded as the seventh most important food crop. However, there has been no comprehensive study of the drought tolerance mechanism of different sweet potato cultivars to date. Here, we studied the mechanism for drought response of seven sweet potato drought-tolerant cultivars using the drought coefficients, physiological indicators and transcriptome sequencing. The seven sweet potato cultivars were classified into four groups of drought tolerance performance. A large number of new genes and transcripts were identified, with an average of about 8000 new genes per sample. Alternative splicing events in sweet potato, which were dominated by first exon and last exon alternative splicing, were not conserved among different cultivars and not significantly affected by drought stress. Furthermore, different drought-tolerance mechanisms were revealed through differentially expressed gene analysis and functional annotation. Two drought-sensitive cultivars, Shangshu-9 and Xushu-22, mainly resisted drought stress by up-regulating plant signal transduction. The other drought-sensitive cultivar Jishu-26 responded to drought stress by down-regulating isoquinoline alkaloid biosynthesis and nitrogen/carbohydrate metabolism. In addition, the drought-tolerant cultivar Chaoshu-1 and drought-preferred cultivar Z15-1 only shared 9% of differentially expressed genes, as well as many opposite metabolic pathways in response to drought. They mainly regulated flavonoid and carbohydrate biosynthesis/metabolism in response to drought, while Z15-1 increased photosynthesis and carbon fixation capacity. The other drought-tolerant cultivar Xushu-18 responded to drought stress by regulating the isoquinoline alkaloid biosynthesis and nitrogen/carbohydrate metabolism. The extremely drought-tolerant cultivar Xuzi-8 was almost unaffected by drought stress and responded to drought environment only by regulating the cell wall. These findings provide important information for the selection of sweet potatoes for specific purposes.

## 1 Introduction

Due to climate fluctuations and irregular rainfall, crops are frequently exposed to various abiotic stresses such as drought, salinity, high temperature and cold, among which drought is a major limiting factor for crop yield (Berger et al., 2016). It has been predicted that future droughts are likely to exceed those of past centuries in duration, severity and frequency (Ault Toby, 2020). Drought affects a series of physiological and biochemical processes such as photosynthesis, respiration, transport, ion uptake and nutrient metabolism. Moreover, the effect of water deficiency on various physiological indicators of plants often varies with the severity and duration of drought (Gajanayake et al., 2014), ultimately inhibiting plant growth and leading to severe yield losses, Therefore, drought has become an important issue in food production to be addressed. In recent decades, great efforts have been made to breed more drought-tolerant plant species by exploring the physiological and biochemical processes and genetic diversity of plant drought resistance (Kholova et al., 2021). Therefore, understanding the mechanism for drought resistance in plants is important for improving the yield of crops under adverse conditions.

Sweet potato, an important food source for humans, is a root crop widely grown in some Asian and African countries (i.e. China, India and Kenya). Due to its high adaptability, nutrient content, stability and yield, low input requirements, versatility and many other advantages (Ahn et al., 2010; Oliveira et al., 2019; Nakagawa et al., 2021), sweet potato is considered as the seventh most important food crop producing a large amount of food per unit area per unit time (Davis et al., 2004). Because sweet potato is generally cultivated on arid and semi-arid lands, drought tolerance is an important target in its breeding. Sweet potato has 90 chromosomes (2n = 6X = 90), with great homogeneity and a genome size of over 2.4 GB (Yang et al., 2017). In addition, the breeding of sweet potato is largely limited by its self- and cross-incompatibility (Gurmu et al., 2013). Therefore, drought-tolerant breeding of sweet potato is confronted with various challenges.

Transcriptome sequencing can rapidly screen the drought tolerance genes and also identify related signaling pathways (Cao et al., 2016). Previous studies used second- and third-generation sequencing technologies as well as the Illumina platform to study the transcriptomes of several sweetpotato species through transcriptome sequencing and *de novo* transcriptome assembly (Wang et al., 2010b; Zhu et al., 2019), and found that *Ipomoea trifida* is the closest wild relative of *Ipomoea batatas*, and may be the ancestor of sweet potato (Roullier et al., 2013). In transcriptome sequencing of sweet potato, researchers have obtained much information about a number of viral infections (Sung et al., 2019; Jo et al., 2020) and abiotic stresses, including traumatic injuries (Kuo et al., 2019), drought and salt stress (Xie et al., 2014), and low temperature stress (Ji et al., 2019; Ji et al., 2020). For drought stress, Lau et al. (2018) screened 122 candidate drought tolerance genes by polyethylene glycol treatment to simulate drought conditions using RNA-Seq. Arisha et al. (2020) studied the differentially expressed genes in leaves of purple-fleshed sweet potato under diffenent drought stresses through thranscriptome sequencing. there have been few studies of the drought tolerance mechanism of different sweet potato varieties under direct water deficiency.

In this study, we used seven sweet potato cultivars with different drought tolerance as materials to reveal the mechanisms for different drought tolerance in sweet potato by analyzing their drought tolerance characteristics using second-generation sequencing technology. Our results provide new insights into the drought tolerance of different sweet potato cultivars and reveal the potential defense mechanisms of specific genes involved in drought tolerance, which may provide some guidance for future breeding of more drought-tolerant sweet potato.

## 2 Materials and methods

### 2.1 Plant materials and cultivation

Sweet potato cultivars were provided by the Sweet Potato Research Institute of the China Agriculture Academy of Science. Seven sweet potato cultivars were classified into different drought resistant types, including Shangshu-9 (S1), Chaoshu-1 (S2), Xushu-22 (S3), Z15-1 (S4), Xushu-18 (S5), Jishu-26 (S6) and Xuzi-8 (S7) sweet potatoes. Sweet potato seedlings of uniform size were selected and planted in pots (800 × 350 × 300 mm) with 2 plants per pot, and the seedlings were acclimatized for 10 d after planting and moved to the greenhouse for moisture treatment after the slow seedling period. Eight pots for each cultivar were planted per treatment, which were equally divided into two groups for drought stress and control treatment, respectively.

Each pot was filled with 30 kg of grass charcoal soil. Three sweet potato plants were planted in each pot. 10.8 g of ammonium dihydrogen phosphate was applied throughout the reproductive period, and the maximum field moisture capacity was 25% (1.4 g/cm^3^ of soil bulk density). Watering was stopped at potato expansion period (about day 35) and drought stress was started. For drought stress, the soil moisture was maintained at about 30% – 40% of the field moisture capacity (stopping watering), and for the control treatment, the soil moisture was kept at about 70% – 80% of the field moisture capacity (normal watering). Soil moisture regulation was conducted by weighing method. The leaves from the the seedlings were collected as samples after 30 days of drought stress for determination of chlorophyll, proline, malonaldehyde (MDA) contents determination, and RNA extraction. Collected samples were frozen in liquid nitrogen and stored at −80 °C for RNA extraction. Each treatment had three biological replicates.

Total chlorophyll content was determined using the equation proposed by Xia et al. (2020). Proline content was estimated according to the method reported by Bates et al. (1973). The proline content was estimated from the standard curve using L-proline and expressed as μg/g of fresh weight. MDA content was estimated according to the method given by Peng et al. (2021) and expressed as μg/g of fresh weight.

### 2.2 Assessment of drought tolerance indices

The trial was conducted at the comprehensive experimental site of Xinjiang Academy of Agricultural Sciences (87.465 N, 43.955 S) and the sweet potato seedlings were planted manually in mid-May 2018 at a planting density of 60,000 plants/hm2. Watering was stopped from day 35 (potato expansion period) and drought stress was started. For the control treatment, the soil moisture was maintained at about 70%–80% of the field moisture capacity (normal watering). Each sample square was planted with 100 plants in three parallels. The yield of fresh sweet potatoes was obtained by plot measurement at harvest, and the yield of fresh sweet potatoes per unit area was calculated (kg/hm^2^). Drought resistance coefficient (DRC) and drought sensitivity index (DSI) were used to determine the tolerance and susceptibility of sweet potatoes (Bouslama and Schapaugh, 1984; Mehrdad et al., 2011).

DRC and DSI were determined as follows: DRC = Y_S_/Y_P_, DSI = (1 − Y_S_/Y_P_)/(1 −_S_/_P_), where,Ys is the yield under drought stress of individual genotypes,Yp is the yield under no drought stress of individual genotypes, _S_ is the mean yield under drought stress, _P_ is the mean yield under no moistures stress. DRC value < 0.7 indicated sensitivity to drought; 0.7< DSI value <0.8 represented tolerance to drought; 0.8< DSI value < 1 meant extreme tolerance to drought, and DSI value > 1 referred to preference to drought. DSI value < 0 indicated preference to drought; 0 < DSI value <0.6 indicated tolerance to drought; 0.6 < DSI value <1 represented extreme tolerance to drought; and DSI value >1 indicated sensitivity.

### 2.3 RNA extraction and cDNA library construction

Total RNA from leaves was extracted by grinding the tissue in TRIZOL reagent. To determine the RNA quality, samples were assessed using a NanoDrop microspectrophotometer (Thermo Fisher Scientific) and an Agilent 2100 Bioanalyzer (Agilent Technologies). The RNA samples were reverse transcribed into cDNA using the SMARTer^®^ PCR cDNA synthesis kit and optimized to prepare cDNA library

### 2.4 Transcriptome sequencing and sequence analysis

The qualified libraries were sequenced by the Illumina NovaSeq 6000 instrument, and all the original sequences were converted into circular consensus sequences (CCS) according to the adaptor in the sequence. Then, the sequences were divided into full-length and non-full-length sequences according to the presence of 3’ primer, 5’ primer and PolyA in CCS sequences. The full-length sequences from the same transcript were clustered, and similar full-length sequences were clustered together, and each cluster was assigned with a consensus sequence. Finally, the non-full-length sequences were corrected (polishing) to obtain high-quality sequences for subsequent analysis

### 2.5 Characterization of alternative splicing events

The determination of alternative splicing (AS) events was carried out using the ASprofile tool (Florea et al., 2013) with default parameters. The AS events were divided into five different types and 12 sub-categories according to the structure of the exon (Florea et al., 2013; Wang et al., 2016). Exons absent in other isoforms were considered exon skipping events (exon skip, ES), including skipped exon (SKIP), approximate skipped exon (XSKIP), Multi-exon skipped exon (MSKIP), and approximate Multi-exon skipped exon (XMSKIP). Introns fully subsumed by an exon were labelled as retained (intron retention, IR), including single intron retention (SIR), approximate intron retention (XIR), Multi-intron retention (MIR) and approximate Multi-intron retention (XMIR). Transcription start site (TSS, or A3) that differed at their 3’ splice junctions were considered as alternative. Transcription terminal site (TTS, or A5) that differed at their 5’ splice junctions were considered alternative. The constitutive exon cannot coexist in the same transcript as mutually exclusive exons (mutually exclusive exon, ME), including alternative exon ends (5’, 3’, or both, AE) and Approximate alternative exon ends (XAE).

### 2.6 Functional annotation

Corrected isoforms were searched against NCBI non-redundant (NR), NCBI nucleotide sequence (NT), Swiss-Prot (a manually annotated and reviewed protein sequence database), Cluster of Orthologous Groups (KOG/COG) (Tatusov et al., 2000) and Kyoto Encyclopedia of Genes and Genomes (KEGG) (Kanehisa et al., 2004) databases with BLAST software. Gene Ontology (GO) annotations were determined based on the best BLASTX hit from the NR database using the Blast2GO software (Götz et al., 2008). KEGG pathway analyses were performed using KOBAS 3.0 software (http://kobas.cbi.pku.edu.cn/index.php) (Mao et al., 2005), and HMMER software was used to search the Pfam database (Mistry et al., 2013). The GO and KEGG pathway enrichment analyses of DEGs were conducted using the R package.

### 2.7 Quantification of gene expression levels and differential expression analysis

Transcriptome sequencing was accomplished based on Illumina sequencing platform, and the number of transcripts per million clean tags (TPM), reads per kilobase per million mapped reads (RPKM), fragments per kilobase of transcript per million fragments mapped (FPKM) and fold change of FPKM were recorded for each replicate of each library separately. Finally, clean and high-quality reads were aligned and mapped to the reference genome of *I. batatas* (cv.Taizhong6, https://sweetpotao.com/download_genome.html). RSEM software was used to compute the FPKM of each gene (Florea et al., 2013). Differentially expressed genes (DEGs) were detected in the different samples according to the fold change (FC) of the FPKM values using DESeq (Wang et al., 2009). A false discovery rate (FDR) control was utilized to calculate the threshold of the P-value. The threshold for the screening of DEGs was set at an absolute value of log_2_ FC ≥ 1 and an FDR significance score less than 0.05.

## 3 Result

### 3.1 Physiological response of different sweetpotato cultivars to drought stress

**Figure 1:**
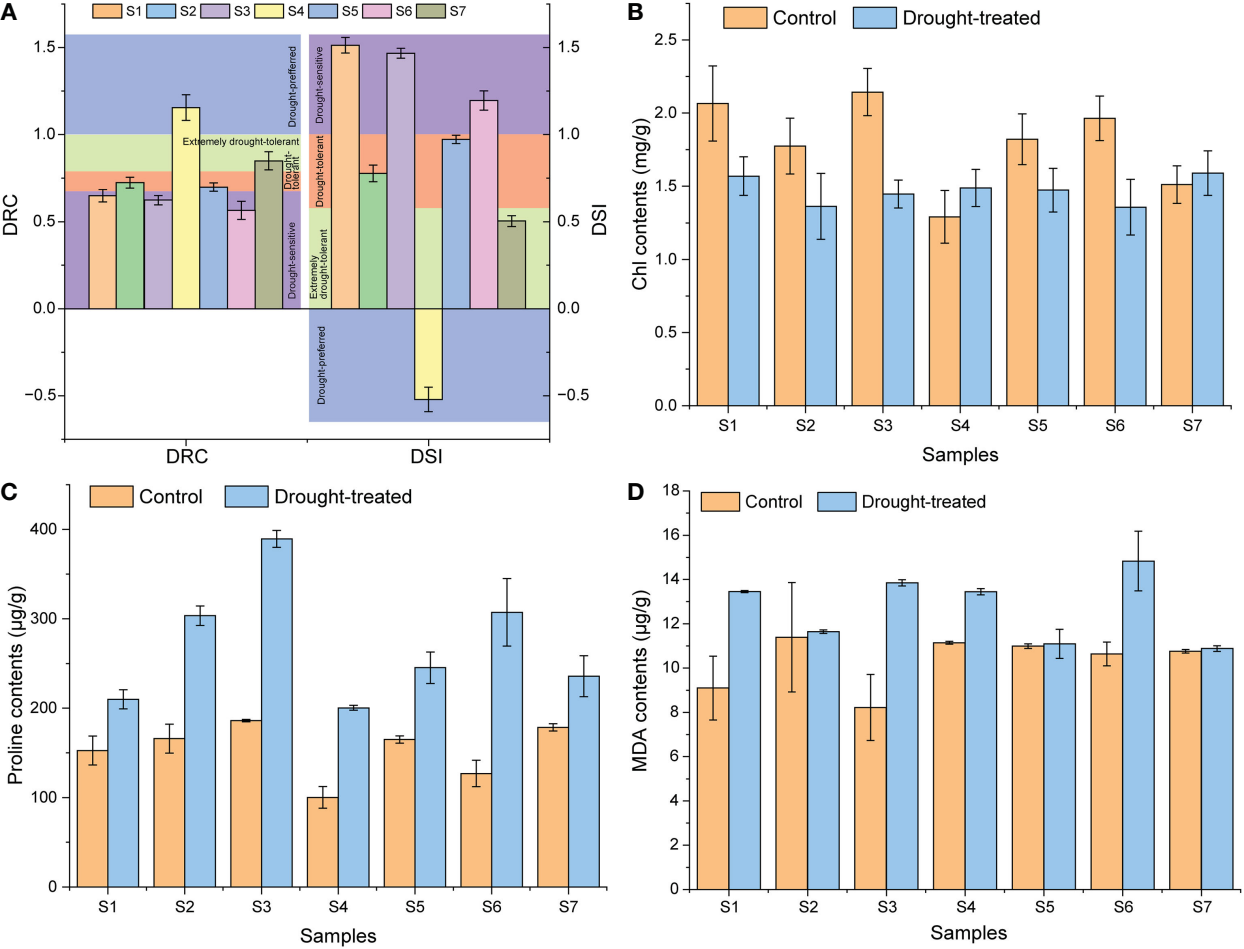
Physiological response of different sweet potato cultivars. **(A)**, the DRC and DSI values; **(B)**, the Chl contents. **(C)**, the proline contents. **(D)**, the MDA contents. S1, Control Shangshu-9; H1, Drought-treated Shangshu-9; S2, Control Chaoshu-1; H2, Drought-treated Chaoshu-1; S3, Control Xushu-22; H3, Drought-treated Xushu-22; S4, Control Z15-1; H4, Drought-treated Z15-1; S5, Control Xushu-18; H5, Drought-treated Xushu-18; S6, Control Jishu-26; H6, Drought-treated Jishu-26; S7, Control Xuzi-8; H7, Drought-treated Xuzi-8.

In order to evaluate the drought resistance characteristics of several cultivars, we firstly studied the physiological response of several cultivars to drought stress. Firstly, based on the DRC and DSI, the seven sweet potato cultivars were classified into four categories (**Figure 1A**). The drought-sensitive cultivars were S1 (Shangshu-9), S3 (Xushu-22), and S6 (Jishu-26); drought-tolerant cultivars included S2 (Chaoshu-1) and S5 (Xushu-18) cultivars; extremely drought-tolerant cultivar was S7 (Xuzi-8) cultivar; and drought-loving cultivar was S4 (Z15-1). In terms of chlorophyll contents, drought-sensitive cultivars showed a significant decrease, while the drought-tolerant cultivars exhibited a less significant decrease, and extremely drought-tolerant and drought-loving cultivars showed no significant change under drought conditions (**Figure 1B**). Proline content increased significantly in all cultivars under drought conditions, but MDA content only increased significantly in drought-sensitive cultivars (**Figures 1C, D**). These physiological responses verified that different sweet potato cultivars have different response sensitivity to drought conditions.

### 3.2 RNA-Seq and *de novo* transcriptome assembly

A total of 293.82 Gb and 6.28 Gb clean data of each sample were generated after the removal of adaptor sequences and low quality reads, respectively. The average sequencing depth per sample was about 23,393,313 clean reads, and drought stress seemed to increase the number of clean reads (**Table S1**). Guanine-cytosine (GC) content ranged from 46%–48% and Q30 ranged from 93%–94%. The ratio of genomic reads to clean reads was all greater than 74%, which was sufficient for *de novo* transcriptome assembly.

A total of 62,882 genes were detected and 56,835 genes were annotated (**Table S2**). New genes were defined as unigenes identified in the sequencing results but not found in the reference genome. Based on the new gene analysis, an average of about 8000 new genes per sample were unique to sweet potato (**Table S3**). Principal coordinate analysis (PCoA) with weighted UniFrac distance was performed to explore and visualize the similarities or differences in genes of different sweet potato cultivars under drought stress. **Figure S1** shows that different sweet potato cultivars could be clustered separately after drought stress, indicating a good experimental setup. Before and after drought stress, S1, S3 and S7 showed shorter distances than S2 and S5. However, S4 and S6 showed the largest variations in genes after drought stress among the seven cultivars.

### 3.3 AS modes with different sweet potato cultivars

**Figure 2:**
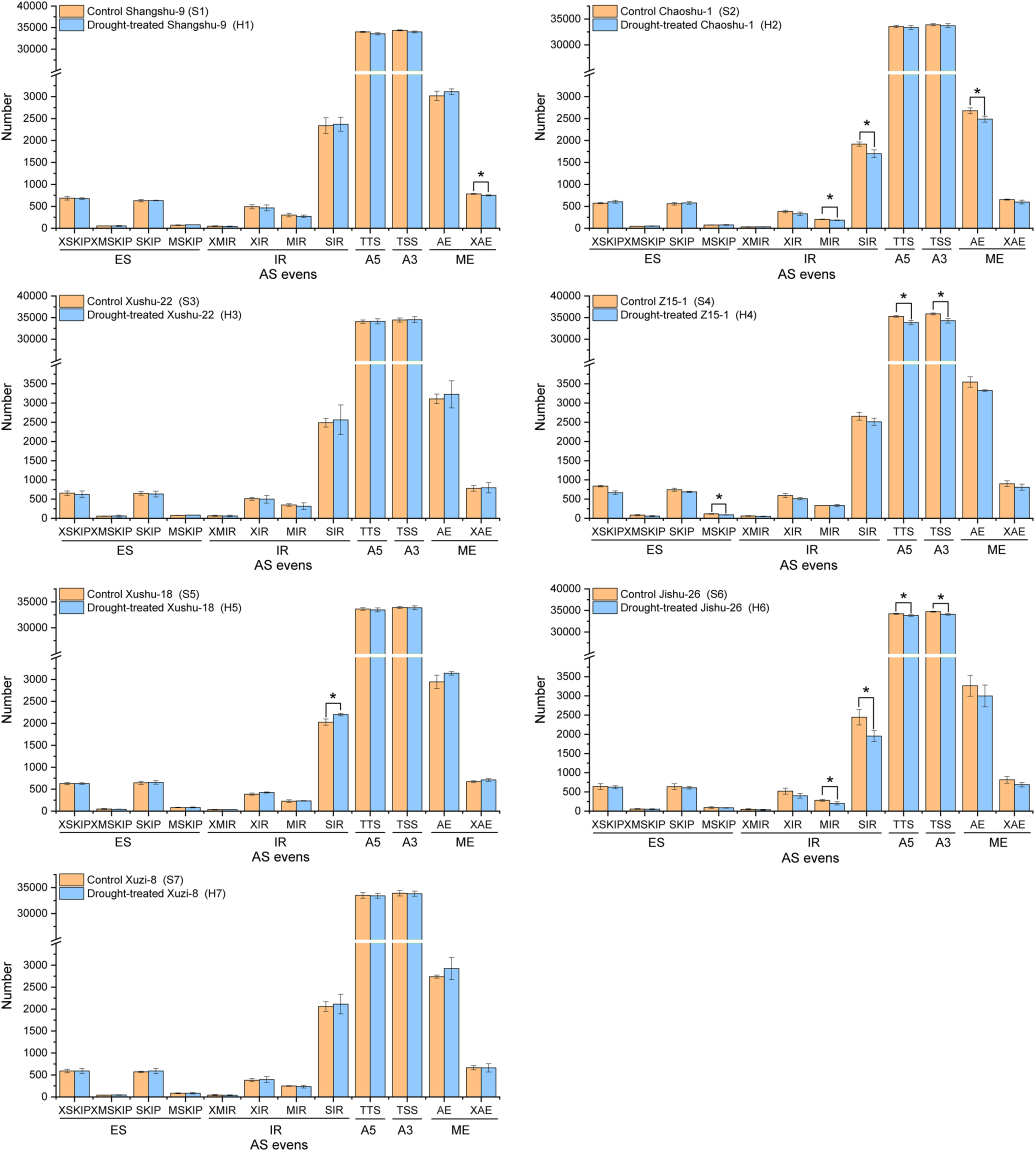
Identification of Alternative splicing (AS) events of different sweet potato cultivars. TSS, transcription start site (or alternative 5’ first exon, A5); TTS, transcription terminal site (or alternative 3’ last exon, A3); SKIP, skipped exon (SKIP_ON, SKIP_OFF pair); XSKIP, approximate SKIP (XSKIP_ON, XSKIP_OFF pair); MSKIP, Multi-exon SKIP (MSKIP_ON, MSKIP_OFF pair);XMSKIP, Approximate MSKIP (XMSKIP_ON, XMSKIP_OFF pair); SIR, Single intron retention (IR_ON, IR_OFF pair); XIR, Approximate IR (XIR_ON, XIR_OFF pair); MIR, Multi-IR (MIR_ON, MIR_OFF pair); XMIR, Approximate MIR (XMIR_ON, XMIR_OFF pair); AE, Alternative exon ends (5’, 3’, or both); XAE, Approximate AE; ES, exon skip, including SKIP, XSKIP, MSKIP and XMSKIP; IR, Intron retention, including SIR, XIR, MIR and XMIR; ME, mutually exclusive exon, including AE and XAE. The symbol * means error bars represent the average of three replicates ± SE (* p < 0.05; **p < 0.01).

Transcriptome sequencing technology can yield long reads without the aid of assembly and provides superior evidence for identifying AS variants. Based on the high-quality full-length isoforms, we systematically analyzed the AS events. Five major AS events including IR, TTS, TSS, AE and MX events and 12 types were identified by customizing a user-friendly program. As shown in **Figure 2**, only S4 showed a significant decrease in the total number of AS events under drought conditions, implying that all sweet potato cultivars had certain drought stress tolerance combined with the results of **Figure 1**. TTS and TSS were the main AS modes in all sweet potato cultivars, followed by IR and AE. In addition, S3 and S7 did not undergo significant AS events under drought stress. Only the number of XAE events significantly decreased in S1 after drought stress. The number of MIR, IR and AE events significantly decreased in S2 after drought stress. Only the number of IR events significantly increased in S5 after drought stress. The number of TTS, TSS, MIR and IR events significantly decreased in S6 after drought stress. Among the differential AS events, all types decreased significantly except for IR in S5, which increased significantly under drought stress (*P*<0.05).

### 3.4 DEGs of different sweet potato cultivars under drought stress

**Figure 3:**
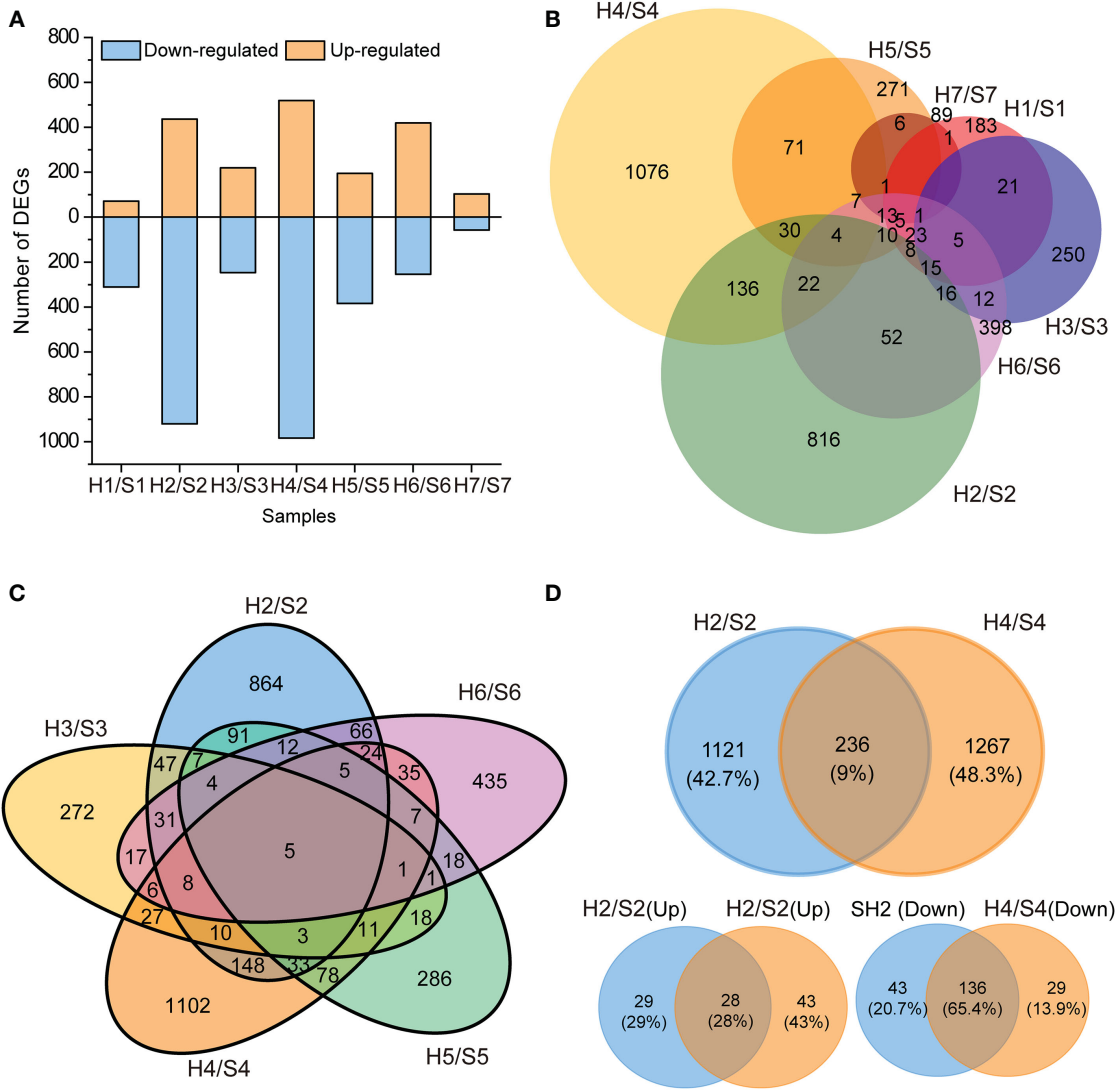
Statistical chart of differentially expressed genes (DEGs) transcriptome in response to drought stress. **(A)**, Number of DEGs (up- and down- regulated). **(B)**, Venn diagram of DEGs in the seven sweet potato cultivars under the drought stress. **(C)**, Venn diagram of DEGs in the five sweet potato cultivars under the drought stress. **(D)**, Venn diagram of DEGs in S2 and S4 sweet potatoes under the drought stress. H1/S1, drought-treated Shangshu-9/control Shangshu-9; H2/S2, drought-treated Chaoshu-1/control Chaoshu-1; H3/S3, drought-treated Xushu-22/control Xushu-22; H4/S4, drought-treated Z15-1/control Z15-1; H5/S5, drought-treated Xushu-18/control Xushu-18; H6/S6, drought-treated Jishu-26/control Jishu-26; H7/S7, drought-treated Xuzi-8/control Xuzi-8.

To quantify the gene expression, the expression of each unigene was calculated by FPKM values, and DEGs were identified using the criteria of log2 FC ≥ 1 in expression during drought stress at a false discovery rate < 0.05. After drought stress, 71, 437, 220, 519, 195, 420, and 104 significantly up-regulated unigenes and 311, 920, 247, 984, 384, 254, and 58 significantly down-regulated unigenes were detected compared with the untreated control (**Figure S2**, **Table S3**). As a whole, the fewest DEGs (only 162) were identified in S7 after drought stress, followed by S1 and S2. By contrast, the largest number of DEGs were identified in S2 and S4 after drought stress. The number of down-regulated genes was greater than that of up-regulated genes in all cultivars except for S6 and S7 (**Figure 3A**; **Table S3**). The total annotation rate could reach 90% by COG, GO, KEGG, KOG, Pfam, Swiss-Prot and Nr, and the annotation rate of all cultivars reached 95% except for S3, which has an annotation rate slightly lower than 95% (**Table S3**).

No common DEGs were found in all seven cultivars after drought stress (**Figure 3B**; **Table S4**), suggesting that these cultivars have different mechanisms for their response to drought stress. No more than 10% of DEGs were shared by S1 and other cultivars, and S1 only shared 1.1% of DEGs with S7 (six DEGs). The largest number of unique DEGs (1076 and 816) was discovered in S1 and S4, respectively, indicating that these two cultivars are the most sensitive to drought stress. The fewest unique DEGs to S7 indicated that this cultivar is the most tolerant to drought stress. Although the largest number of DEGs was identified in S2 and S4 after drought stress, the DEGs shared by them was only 9% (236 DEGs) of the total. Among the 236 shared DEGs, 65.4% (136) shared DEGs were down-regulated specifically, and 28% (28) were up-regulated under drought stress (**Figure 3D**). Moreover, 12.1% of DEGs (255 DEGs) were shared by S2 and S5 (**Figure 3C**).

### 3.5 Functional annotation of DEGs in sweet potato cultivars

**Figure 4:**
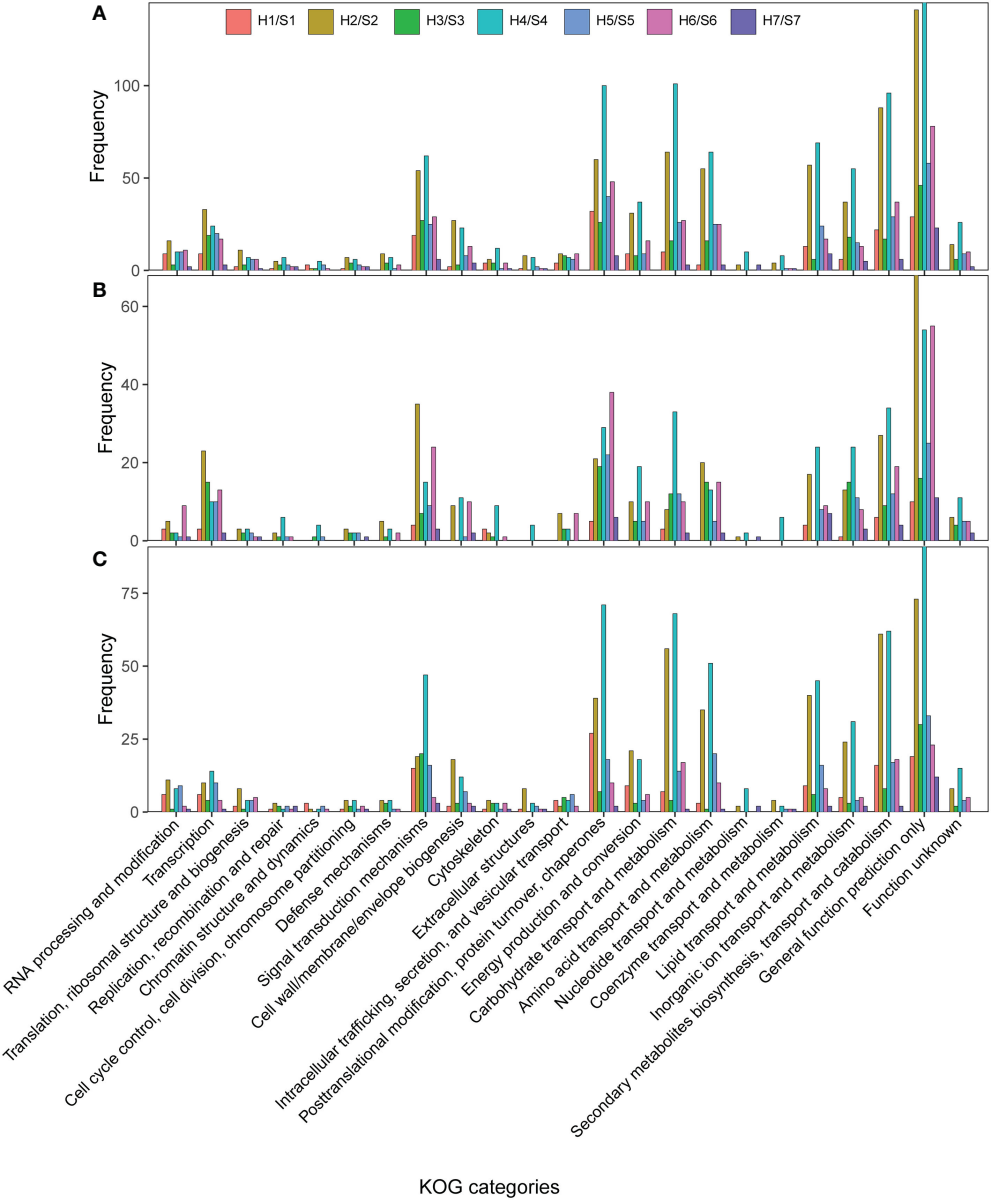
KOG categories of DEGs in the different of Sweet potato cultivars under the drought stress. **(A)**, KOG categories of all DEGs; **(B)**, KOG categories of up-regulated DEGs; **(C)** KOG categories of down-regulated DEGs. H1/S1, drought-treated Shangshu-9/control Shangshu-9; H2/S2, drought-treated Chaoshu-1/control Chaoshu-1; H3/S3, drought-treated Xushu-22/control Xushu-22; H4/S4, drought-treated Z15-1/control Z15-1; H5/S5, drought-treated Xushu-18/control Xushu-18; H6/S6, drought-treated Jishu-26/control Jishu-26; H7/S7, drought-treated Xuzi-8/control Xuzi-8.

The KOG enrichment results of DEGs (**Figure 4**) revealed that a large number of drought-responsive genes identified in different cultivars were involved in signal transduction mechanism, posttranslational modification, protein turnover, chaperones, carbohydrate transport and metabolism, secondary metabolite biosynthesis, transport and catabolism, and lipid transport and metabolism under drought stress. This could be mainly ascribed to drought-responsive genes in S2 and S4 cultivars, particularly S4. In drought-sensitive cultivars, the drought-responsive genes in S1 were mainly involved in posttranslational modification through down-regulation of DEGs; the drought-responsive genes in S3 were mainly involved in signal transduction mechanisms, while those in S6 were involved in carbohydrate transport and metabolism, secondary metabolites biosynthesis, transport and catabolism. The up-regulated drought-responsive genes in S6 were involved in protein turnover, chaperones, and signal transduction mechanisms, while the down-regulated genes were involved in carbohydrate transport and metabolism. In drought-tolerant S2 and S5, the up-regulated drought-responsive genes in S2 were involved in signal transduction mechanisms, while the down-regulated genes were involved in carbohydrate, lipid and amino acid transport and metabolism, posttranslational modification, protein turnover, chaperones, and secondary metabolite biosynthesis, transport and catabolism. Notably, the highest proportion of up-regulated DEGs involved in signal transduction was found in S2 compared with other cultivars. The up-regulated drought-responsive genes in S5 were involved in protein turnover and chaperones. However, the drought-responsive genes in extremely drought-tolerant S7 were involved in many KOG categories. The up- and down-regulated drought-responsive genes in S4 were in involved in KOG categories, which was similar to the down-regulated genes in S2. That is, the same KOG categories were enriched by up-regulated and down-regulated genes in S4. Moreover, S4 had the highest proportion of up-regulated genes in carbohydrate transport and metabolism compared with other cultivars.

**Figure 5:**
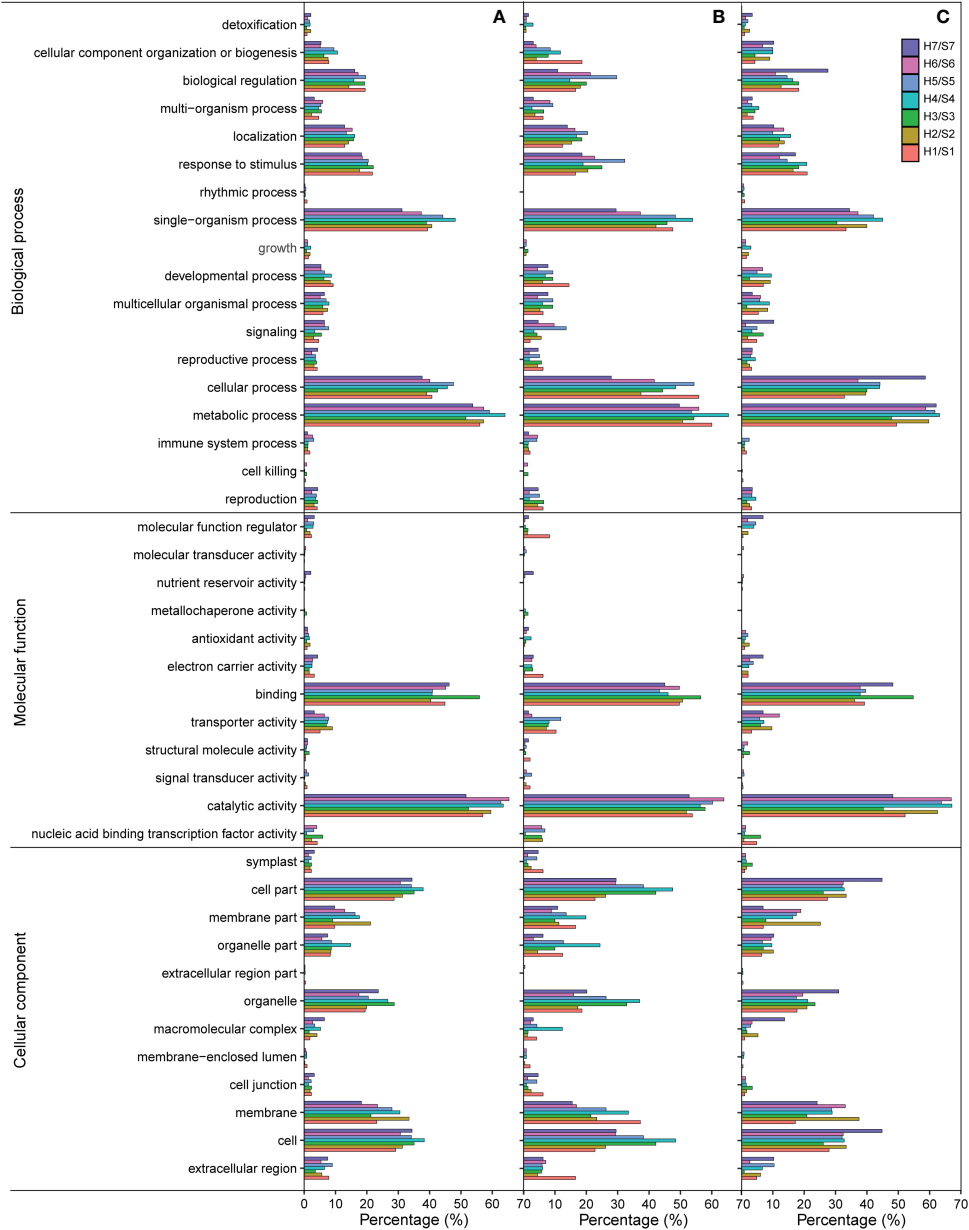
GO terms of DEGs in the different of sweet potato cultivars under the drought stress. **(A)**, GO terms of all DEGs; **(B)**, GO classifications of up-regulated DEGs; **(C)**, GO terms of down-regulated DEGs. H1/S1, drought-treated Shangshu-9/control Shangshu-9; H2/S2, drought-treated Chaoshu-1/control Chaoshu-1; H3/S3, drought-treated Xushu-22/control Xushu-22; H4/S4, drought-treated Z15-1/control Z15-1; H5/S5, drought-treated Xushu-18/control Xushu-18; H6/S6, drought-treated Jishu-26/control Jishu-26; H7/S7, drought-treated Xuzi-8/control Xuzi-8.

The GO clustering analysis of DEGs resulted in three major categories: cellular component (CC), biological process (BP), and molecular function (MF). In the BP category, many DEGs identified in different drought-tolerant cultivars under drought stress were significantly enriched in cellular process and metabolic process, single-organism process, response to stimulus, and biological regulation. During the single-organism process, the proportion of both up-regulated and down-regulated genes in S4 was higher than that in other cultivars. Moreover, during the response to stimulus and biological regulation, the proportion of up-regulated genes in S5 was significantly higher than that in other cultivars, with the highest proportion of down-regulated genes being found in S7 during biological regulation (**Figure 5**). In the MF category, the most abundant genes were found to be involved in the binding, where the highest proportion of both up-regulated and down-regulated genes was present in S3, and catalytic activity, where the highest proportion of both up-regulated and down-regulated genes was found in S6. In the CC category, the most abundant genes were involved in the cell, cell part, membrane, membrane part, and organelle. The proportion of up-regulated genes in the cell membrane fraction was higher in S1 than in other cultivars, and that of down-regulated genes in the cell fraction was the highest in S7.

**Figure 6:**
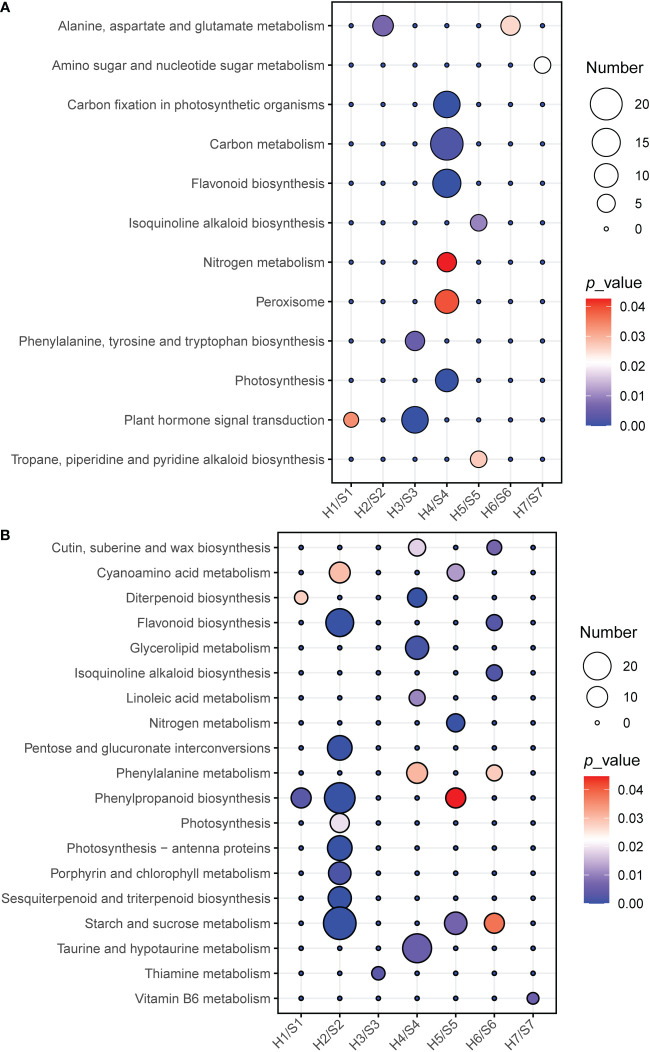
KEGG enrichment analysis of DEGs in the different of sweet potato cultivars under the drought stress. **(A)**, KEGG enrichment of up-regulated DEG, statistics of pathway enrichment (*P*<0.05), **(B)**, KEGG enrichment of down-regulated DEG, statistics of pathway enrichment (*P <*0.05). The number of DEG is distinguished by the size of the circle and the circle from blue to red represents the P-value from large to small. H1/S1, drought-treated Shangshu-9/control Shangshu-9; H2/S2, drought-treated Chaoshu-1/control Chaoshu-1; H3/S3, drought-treated Xushu-22/control Xushu-22; H4/S4, drought-treated Z15-1/control Z15-1; H5/S5, drought-treated Xushu-18/control Xushu-18; H6/S6, drought-treated Jishu-26/control Jishu-26; H7/S7, drought-treated Xuzi-8/control Xuzi-8.

KEGG enrichment results (**Figure 6**; **Table S5**) showed that a large number of pathways were significantly down-regulated under drought stress, suggesting that drought is a hazardous environmental stress for most of these cultivars. Plant hormone signal transduction was significantly up-regulated enriched in S1 and S3. Alanine, aspartate and glutamate metabolisms were significantly up-regulated enriched in S2 and S6. Phenypropanoid biosynthesis was significantly down-regulated enriched in S1, S2 and S5. Flavonoid biosynthesis was significantly down-regulated enriched in S1, S2 and S6. Starch and sucrose metabolism was significantly down-regulated enriched in S2, S4 and S6. S7 was only enriched in up-regulated metabolism of amino sugars and nucleotide sugars and down-regulated Vitamin B6 metabolism, with only four up-regulated and two down-regulated genes, indicating that drought has no significant effect on its growth or metabolic functions. Notably, in contrast to other cultivars, S4 not only has higher photosynthesis and carbon fixation capacity but also stronger resistance to the generation of reactive oxygen by up-regulating flavonoid synthesis and peroxisomes, thereby avoiding cellular oxidative damage under drought stress. Particularly, S4 showed opposite behaviors to S2 in many metabolic pathways, especially for photosynthesis. For example, flavonoid biosynthesis was down-regulated in S2 but up-regulated in S4.

## 4 Discussion

Sweet potato is a rich source of nutrients. However, increases in the degree and frequency of drought largely hinder the sustainable production of sweet potato. Considering the severity of drought stress and the complexity of the sweet potato genome, this study used transcriptome sequencing technologies to reveal the mechanisms of drought stress tolerance in different drought-tolerant cultivars, which may further promote the breeding of drought-tolerant sweet potato cultivars

### 4.1 Characteristics of different sweet potatoes cultivars

The seven cultivars studied here had different drought tolerance performance. DRC and DSI were used to evaluate the drought resistance of different sweet potato cultivars (Kivuva et al., 2015). Zhou et al. (2016) have reported the drought tolerance indices of Shangshu-9 (S1, better disease resistance), Chaoshu-1 (S2), Xushu-22 (S3, wide adaptability), Xushu-18 (S5, drought tolerance) and Jishu-26 (S6, drought and barrenness tolerance). The highest value was 1.25 for S1, followed by 0.98 and 0.97 for S2 and S3, respectively, and the lowest value was 0.65 for S5, and the value was close to 0.74 for S6. Some studies have reported that both S5 and S6 are of medium drought tolerance (Zhang et al., 2022a). Moreover, the transcriptome results of flowering under drought stress indicated that S5 is drought tolerant (Tao et al., 2013). There has been no report about the drought tolerance index of the other two sweet potato cultivars. Z15-1 (S4) is tolerant to barrenness and can withstand nutrient stress. Xuzi-8 (S7) is a drought tolerant and early maturing sweet potato with high antioxidant capacity due to its high anthocyanin content (Arisha et al., 2020). Therefore, these sweet potatoes cultivars have certain drought tolerance. In this study, S1, S3 and S6 were classified as relatively drought-sensitive cultivars, which does not mean that they are not drought tolerant at all, but just less tolerant than other cultivars. Based on the results of DEGs and enrichment analysis, S4 may be a special cultivar, while S7 is an extremely drought-tolerant cultivar. However, little research has been reported on the mechanism of drought tolerance of different sweet potato cultivars, and Yu et al. (2016) showed that S3 can tolerate 100 mM NaCl stress through changing ion homeostasis and nitrogen metabolism.

### 4.2 Complexity of AS under drought stress

AS is involved in most plant processes and particularly prevalent in plants when exposed to environmental stress during development, in flowering time control, and in the circadian timing system (Wang and Brendel, 2006; Staiger and Brown, 2013). AS is also important in responding to drought. Many studies have shown that AS events are heavily induced in drought response, Song et al, 2020 shown that soybean (Glycine max) roots can respond to different levels of drought stress through differential AS regulation. Drought response is also present in the AS regulation of responsive genes. For example, Os DREB2B2 of rice was significantly induced by drought in two AS events, resulting in enhancement of drought tolerance. Similar AS changes have been reported in wheat (*Triticum aestivum*), and maize (Zea mays). These studies emplasized the conserved pattern of AS regulation among plant species (Matsukura et al., 2010; Qin et al., 2007; Terashima and Takumi, 2009). However, AS response to drought differed in different rice cultivars (Wei et al., 2017). Additionally, homologs of wheat showed different AS responses under stress conditions (Liu et al., 2018). Meanwhile, IR AS is generally dominant in plants (Li et al., 2017; Yao et al., 2020). In this study, TTS and TSS events toether accounted of nearly 90% of the events, and this proportion significantly different from other plants such as Zea mays and cotton (*Gossypium* spp.) (Thatcher et al., 2014; Wang et al., 2018). The number of AS events is high in sweet potato and varies among different cultivars (**Figure 2**). Moreover, different AS modes in different cultivars after drought stress, indicating that AS modes is not very conserved in different sweet potato cultivars and less affected by drought stress.

### 4.3 Mechanisms of drought tolerance in different sweet potato cultivars

**Figure 7:**
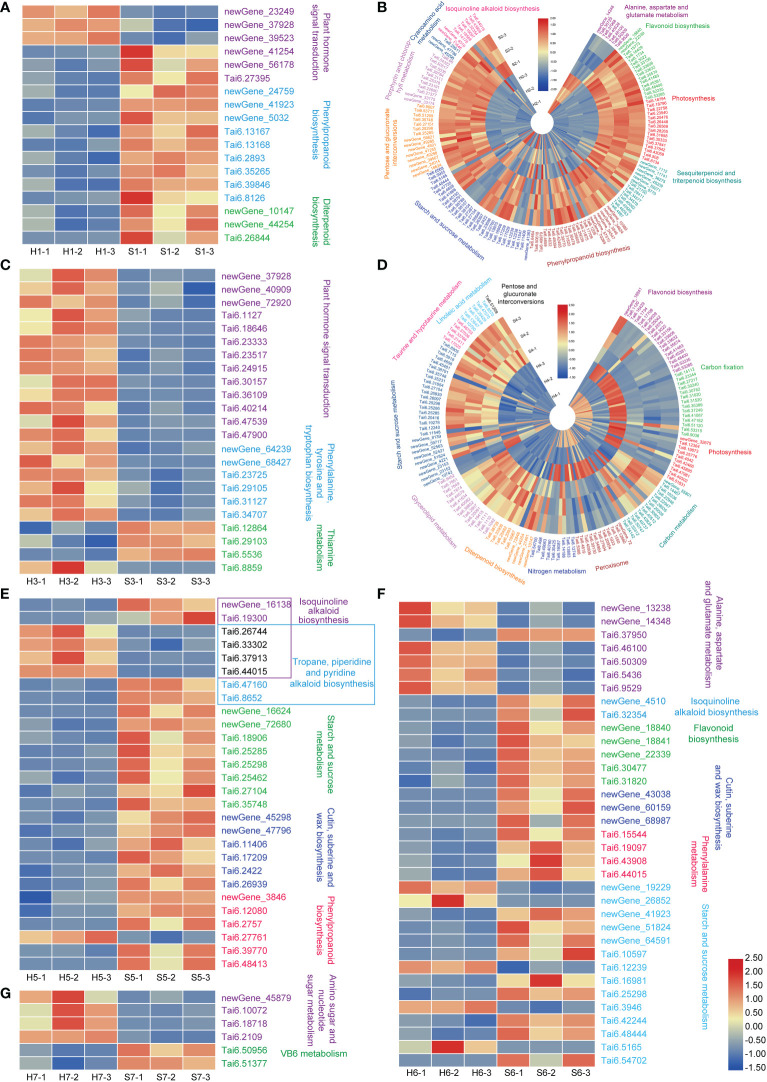
Heatmaps of the enriched KEGG pathways of DEGs in the different of sweet potato cultivars under the drought stress. **(A)**, drought-treated Shangshu-9/control Shangshu-9; **(B)**, drought-treated Chaoshu-1/control Chaoshu-1; **(C)**, drought-treated Xushu-22/control Xushu-22; **(D)**, drought-treated Z15-1/control Z15-1; **(E)**, drought-treated Xushu-18/control Xushu-18; **(F)**, drought-treated Jishu-26/control Jishu-26; **(G)**, drought-treated Xuzi-8/control Xuzi-8.

The response mechanisms of different sweet potato cultivars to drought were clearly represented by the DEGs, annotation of KOG categories, GO terms, and the enrichment of significantly different KEGG pathways. S7 had only 162 DEGs, and showed only one up-regulated metabolic pathway and one down-regulated metabolic pathway, indicating that this cultivar is hardly affected by drought stress. The up-regulated metabolic pathways are the amino sugar and nucleotide sugar metabolism, mainly including the uridine diphosphate (UDP)-glucose synthesis pathway (newGene_45879, encoding UDP-arabinopyranose mutase; Tai6.10072 encoding ADP-glucose pyrophosphorylase; Tai6.18718, encoding Glucose-1-phosphate adenylyltransferase large subunit 1; and Tai6.2109) (**Figure 7**), suggesting that drought stress can only affect the pathways related to cell wall or starch accumulation in S7.

Adverse abiotic stresses tend to elicit multi-level responses, involving stress sensing, signal transduction, transcription, transcript processing, translation and post-translational protein modification (Zhang et al., 2022b). In this study, S1 and S3 showed induced transcriptional expression of related genes through up-regulation of plant hormone signal transduction pathways to resist drought stress, and three genes (newGene_23249, newGene_37928, and newGene_39523, **Figure 7**) in S1 encoded type 2C protein phosphatase (PP2C) with an important partner of abscisic acid (ABA), which negatively regulate ABA signaling and stress responses (Zhang et al., 2008). In plants, ABA is accumulated under osmotic stress caused by drought, and plays a key role in stress response and tolerance (Nakashima et al., 2014). ABA binds to its receptor proteins (pyrabactin resistance/pyr1-like/regulatory family of small soluble protein) and relieves the inhibition of kinase SnRK2 activity by PP2C, thereby inducing a plant stress response. Vranová et al. (2000) reported that the expression of *NtPP2C1* in tobacco was strongly induced by drought and inhibited by oxidative stress and heat shock. However, ABA signaling and drought stress response were regulated by inhibition of *PP2CA* activity in *Arabidopsis thaliana* (Baek et al., 2018). These findings suggest that PP2C may constitute a convergence point in response to adversity. The expression of genes encoding auxin/indoleacetic acids proteins (Aux/IAAs) was mainly up-regulated in S3. AUX/IAA is an important protein transcription factor widely involved in auxin-mediated plant response as well as stress and defense responses, suggesting that AUX/IAA genes respond to drought stress and improve drought resistance in plants. Aux/IAA genes were reported to be involved in regulating drought tolerance in Arabidopsis (*AtIAA5/AtIAA6/AtIAA19*), Sorghum bicolor (*SbIAA8, SbIAA11, SbIAA22, SbIAA23*), rice (*OsIAA6, OsIAA20*) (Wang et al., 2010a; Jung et al., 2015; Salehin et al., 2019; Zhang et al., 2021). In this study, most Aux/IAA genes were up-regulated, particularly Tai6.18646, Tai6.24915 and Tai6.40214 (**Figure 7**).

Secondary metabolite biosynthetic pathways play an important regulatory role in plant resistance to stress. In our research, most genes of the secondary metabolic pathways were enriched in S2 and S4 under drought stress (**Figure 4**). These genes are mainly involved in regulating sesquiterpenoid, triterpenoid, flavonoid, cutin, suberine, wax and isoquinoline alkaloid biosynthesis.

Both the shared DEGs (**Figure 3**) and enriched metabolic pathway in S2 and S4 showed opposite patterns. For example, photosynthesis and carbon metabolism, as well as flavonoid biosynthesis were down-regulated in S2 under drought stress, but it was the opposite for S4. For S4, photosynthesis and carbon fixation genes (glyceraldehyde-3-phosphate dehydrogenase, GAPA; Phosphoribulokinase, PRK), and flavonoid biosynthesis were significantly up-regulated under drought stress, indicating that S4 prefers to drought stress. In particular, 15 genes of flavonoid biosynthesis were up-regulated in S4, while were down-regulated in S2. Anthocyanins, a vital subclass of flavonoids, have antioxidant capacity and can change the color of the root skin and leaf vein base by modulating the flavonoids (Zhao et al., 2022). In this study, flavonoids were increased in S4 under drought stress (8-fold up-regulation of naringenin 3-dioxygenase and Tai6.52235), but decreased in S2 (97-fold down-regulation of Tai6.52235). These results indicate that S2 and S4 mainly regulate the biosynthetic pathways of sesquiterpenoid, triterpenoid and flavonoids in response to drought stress. Additionally, drought stress stimulates a N-mediated tandem reaction in S4, improving its drought tolerance, which is similar to the response to drought stress of Xushu 32 and Ningzishu 1 (Xia et al., 2020).

Isoquinoline alkaloid biosynthesis produces alkaloids, which is indispensable for plant defense against pathogenic infections. The copper-containing amine oxidase (CuAO) is a kind of amine oxidase with various physiological functions, which is involved in plant cell differentiation and response to abiotic stress. Bharalee et al. (2012) found that the induced CuAO gene expression was significantly higher than that of the control under drought conditions, which could improve the resistance of tea to abiotic stress and prevent the accumulation of reactive oxygen species caused by drought. In this article, isoquinoline alkaloid biosynthesis was significantly up-regulated in S5, with an about 2-fold up-regulation of Tai6.33302 and Tai6.44015 genes (encoding CuAO). Moreover, starch, sucrose, and cyanoamino acid metabolism were down-regulated. However, S6 adopted the opposite strategy, in which the isoquinoline alkaloid biosynthesis (mainly polyphenol oxidase, PPO) was down-regulated under drought stress. PPO is considered to be closely related to some specialized pigment biosynthesis and secondary metabolite biosynthesis, and is associated with the down-regulation of flavonoid, suberine and wax biosynthesis pathway in this cultivar. Besides, drought induced biological pathways closely related to alanine, aspartate and glutamate metabolism in S6. Certainly, the role of amino acids in plants cannot be ignored, as they play an assisting role in the biosynthesis of many important metabolites in addition to responding to adverse stresses. Zandalinas et al. (2018) suggested that the main roles of amino acid accumulation in drought environments are protein biosynthesis, recovery after adverse stress, and osmoprotective activity. Changes in these metabolic pathways are central to the metabolism of nitrogen and carbohydrates in S5 and S6, providing a possible explanation for their drought tolerance.

## 5 Conclusion

**Figure 8:**
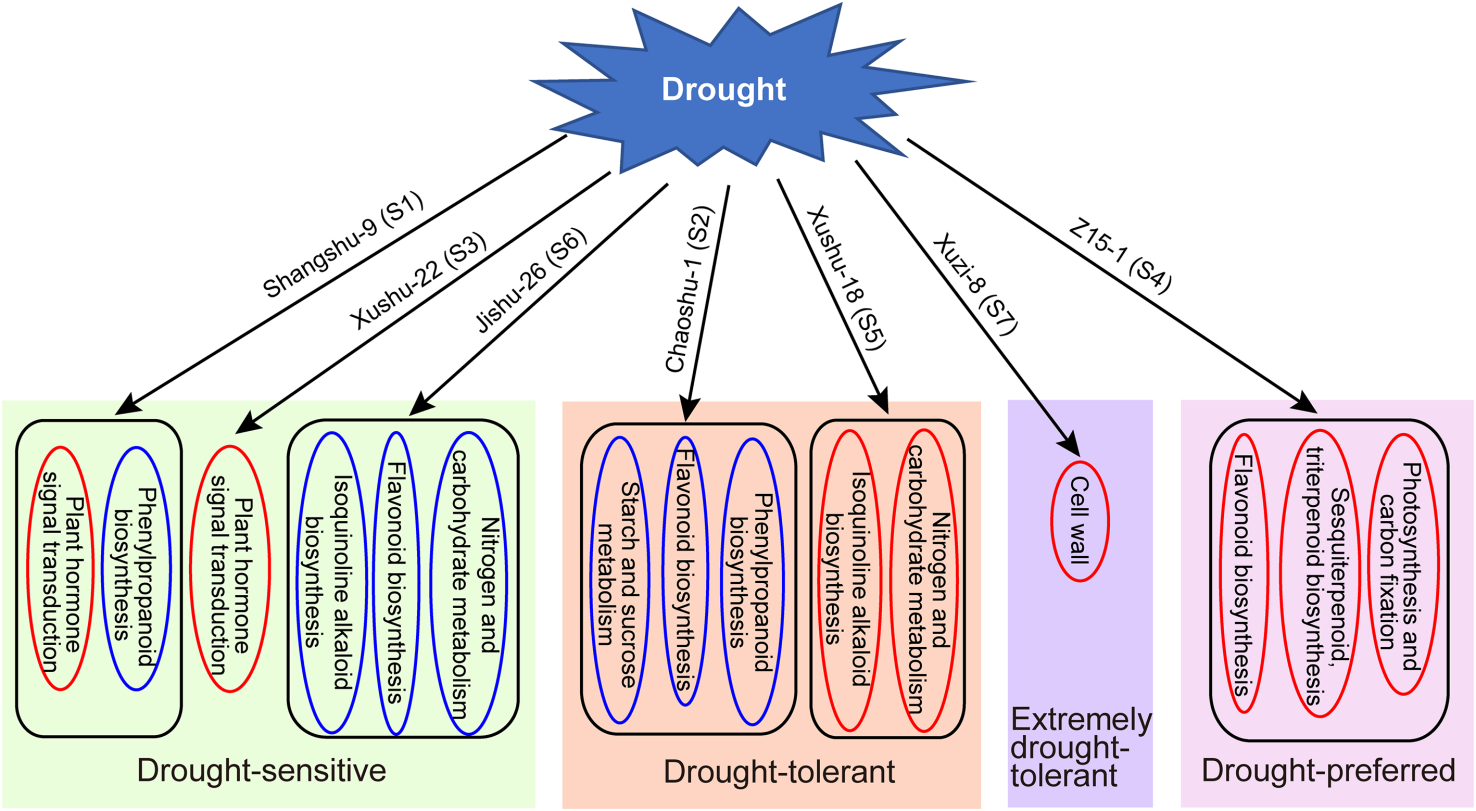
A corresponding working model of different drought tolerant sweet potato clutivars in response to drought. Red box indicate up-regulation, blue box indicate down-regulation.

This study demonstrates the variations in physiological indices and transcriptional alterations of drought-tolerant sweet potato cultivars in response to drought. Based on the results, a corresponding working model was proposed in **Figure 8**. Plant signal transduction, flavonoid biosynthesis, phenypropanoid biosynthesis and isoquinoline alkaloid biosynthesis play important roles in the regulation of drought stress tolerance. In addition, the response mechanism differs very much for different sweet potato cultivars, and is even completely opposite in some cultivars such as Chaoshu-1 and Z15-1 cultivars. Thus, the drought tolerance of sweet potato can be enhanced by these pathways. The results prove the great potential of sweet potato germplasm and provide valuable insights into the drought response mechanisms of sweet potato.

## Data availability statement

The datasets presented in this study can be found in online repositories. The names of the repository/repositories and accession number(s) can be found in the article/**Supplementary Material**.

## Author contributions

PJ and ZZ conceived and designed the experiments. EL, LX performed the experiments and analyzed data. ZLu, ZLi, and GZ contributed reagents/materials/analysis tools. HG, FF, JT, YZ wrote the paper. All authors contributed to the article and approved the submitted version.
