# Supplementary material for: Transcriptomic analysis reveals mechanisms for the different drought tolerance of sweet potatoes
Source: Front Plant Sci. 2023 Mar 16;14:1136709. doi: 10.3389/fpls.2023.1136709 (PMC10060965; doi:10.3389/fpls.2023.1136709)
Supplement: Supplementary file 4 [file DataSheet_1.docx]

Supplementary Material

Transcriptomic analysis reveals mechanisms for the different drought tolerance of sweet potatoes

Enliang Liu**^1†^**, Linli Xu**^2†^**, Zhengqian Luo^2^, Zhiqiang Li^1^, Guohui Zhou^5^, Haifeng Gao^4^, Furong Fang^1^, Jun Tang^3^, Yue Zhao^1^, Zhilin Zhou^3*^, Ping Jin**^2*^**

*** Correspondence:** Ping Jin: [jinp618888@163.com](mailto:jinp618888@163.com)

Zhilin Zhou: [zhilinzhou@jaas.ac.cn](mailto:zhilinzhou@jaas.ac.cn)

# Supplementary Data

# Supplementary Figures and Tables

Figure S1 Principal co-ordinates analysis (PCoA) of the RNA sequencing data of sweet potato cultivars. Each colour represents a biological replicate. S1, Control Shangshu-9; H1, Drought-treated Shangshu-9; S2, Control Chaoshu-1; H2, Drought-treated Chaoshu-1; S3, Control Xushu-22; H3, Drought-treated Xushu-22; S4, Control Z15-1; H4, Drought-treated Z15-1; S5, Control Xushu-18; H5, Drought-treated Xushu-18; S6, Control Jishu-26; H6, Drought-treated Jishu-26; S7, Control Xuzi-8; H7, Drought-treated Xuzi-8.

Figure S2 Volcano plots of DEGs among different cultivars under drought conditions. Red and green dots represent the upregulated and downregulated DEGs, respectively, and black dots represent non-DEGs. H1/S1, drought-treated Shangshu-9/control Shangshu-9; H2/S2, drought-treated Chaoshu-1/control Chaoshu-1; H3/S3, drought-treated Xushu-22/control Xushu-22; H4/S4, drought-treated Z15-1/control Z15-1; H5/S5, drought-treated Xushu-18/control Xushu-18; H6/S6, drought-treated Jishu-26/control Jishu-26; H7/S7, drought-treated Xuzi-8/control Xuzi-8.

Table S1 Next generation sequencing statistical summary of sequenced and assembled results.

Table S2 All Database annotation of different sweet potato cultivars under drought condition.

Table S3 Overview of up-regulated or down-regulated genes in different sweet potato cultivars under normal and drought conditions at a level of |log2 (fold change)| > 1 and FDR < 0.05.

Table S4 The result of Venn.

Table S5 The result of KEGG enrichment in different sweet potato cultivars under drought condition

## Supplementary Figures


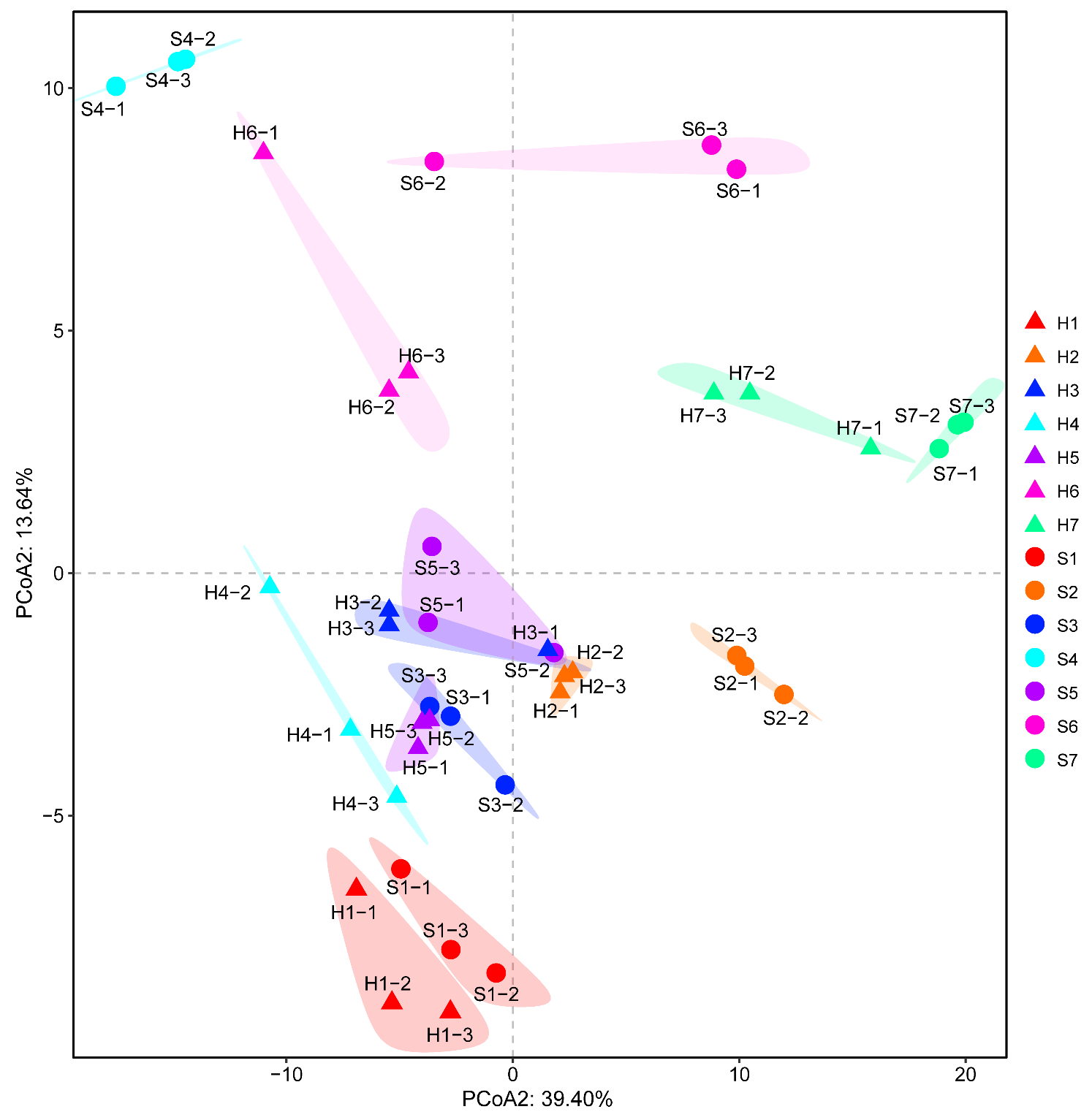


**Supplementary Figure S1.** Principal co-ordinates analysis (PCoA) of the RNA sequencing data of sweet potato cultivars. Each colour represents a biological replicate. S1, Control Shangshu-9; H1, Drought-treated Shangshu-9; S2, Control Chaoshu-1; H2, Drought-treated Chaoshu-1; S3, Control Xushu-22; H3, Drought-treated Xushu-22; S4, Control Z15-1; H4, Drought-treated Z15-1; S5, Control Xushu-18; H5, Drought-treated Xushu-18; S6, Control Jishu-26; H6, Drought-treated Jishu-26; S7, Control Xuzi-8; H7, Drought-treated Xuzi-8.


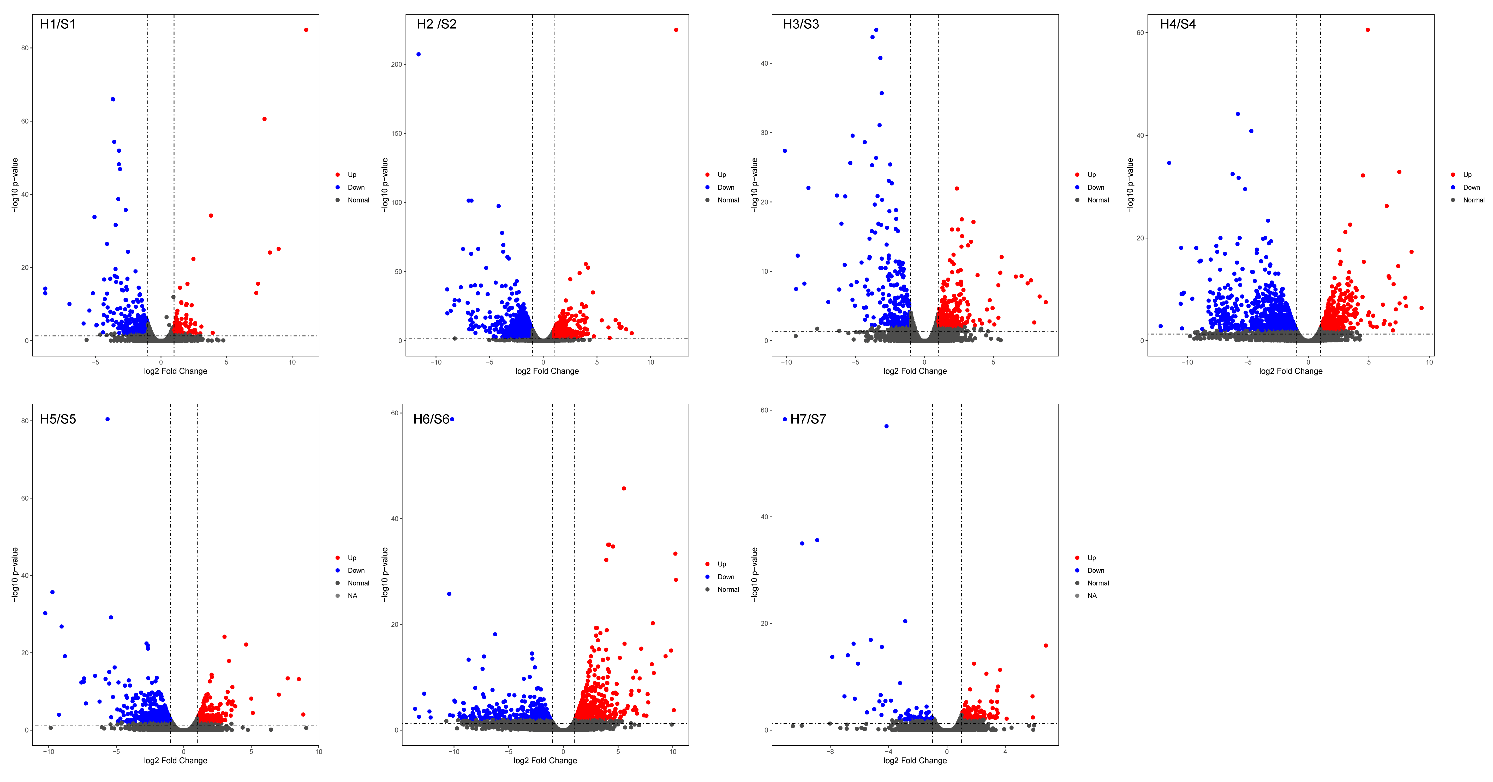


**Supplementary Figure S2.** Volcano plots of DEGs among different cultivars under drought conditions. Red and green dots represent the upregulated and downregulated DEGs, respectively, and black dots represent non-DEGs. H1/S1, drought-treated Shangshu-9/control Shangshu-9; H2/S2, drought-treated Chaoshu-1/control Chaoshu-1; H3/S3, drought-treated Xushu-22/control Xushu-22; H4/S4, drought-treated Z15-1/control Z15-1; H5/S5, drought-treated Xushu-18/control Xushu-18; H6/S6, drought-treated Jishu-26/control Jishu-26; H7/S7, drought-treated Xuzi-8/control Xuzi-8.

## Supplementary Tables

Table S1 Next generation sequencing statistical summary of sequenced and assembled results.

|  | Total Reads | Clean reads | Mapped reads (%) | Clean bases | GC (%) | Q30 (%) |
| --- | --- | --- | --- | --- | --- | --- |
| H1 | 48,354,898.67 ± 834,793.97 | 24,177,449.33 ± 417,396.99 | 74.66% ± 0.24% | 7,239,172,450 ± 125,158,980.54 | 46.95% ± 0.13% | 93.47% ± 0.12% |
| H2 | 44,715,368.00 ± 3,345,906.93 | 22,357,684.00 ± 1,672,953.47 | 74.32% ± 0.45% | 6,691,117,115.33 ± 497,990,899.74 | 47.08% ± 0.11% | 93.59% ± 0.18% |
| H3 | 47,694,194.00 ± 10,191,561.89 | 23,847,097.00 ± 5,095,780.95 | 75.40% ± 0.54% | 7,127,162,318.00 ± 1,506,198,361.25 | 46.94% ± 0.39% | 93.90% ± 0.35% |
| H4 | 42,654,251.33 ± 1,720,400.54 | 21,327,125.67 ± 860,200.27 | 75.07% ± 0.29% | 6,385,742,228.67 ± 256,593,881.92 | 46.61% ± 0.18% | 93.97% ± 0.08% |
| H5 | 45,181,920.67 ± 3,665,611.73 | 22,590,960.33 ± 1,832,805.86 | 75.19% ± 0.29% | 6,764,837,322.67 ± 547,678,042.74 | 47.07% ± 0.12% | 93.69% ± 0.07% |
| H6 | 43,366,758.67 ± 1,613,041.57 | 21,683,379.33 ± 806,520.78 | 73.81% ± 0.16% | 6,489,119,430.00 ± 239,168,128.21 | 46.86% ± 0.17% | 93.73% ± 0.12% |
| H7 | 45,956,990.67 ± 4,492,001.81 | 22,978,495.33 ± 2,246,000.90 | 74.97% ± 0.14% | 6,878,979,326.67 ± 668,407,900.91 | 47.69% ± 0.41% | 94.10% ± 0.37% |
| S1 | 50,198,279.33 ± 3,086,637.55 | 25,099,139.67 ± 1,543,318.78 | 74.08% ± 0.22% | 7,495,947,134.67 ± 440,906,070.52 | 47.14% ± 0.27% | 93.30% ± 0.12% |
| S2 | 45,381,830.00 ± 2,640,994.04 | 22,690,915.00 ± 1,320,497.02 | 74.23% ± 0.02% | 6,784,561,086.00 ± 388,300,111.09 | 47.67% ± 0.10% | 93.46% ± 0.09% |
| S3 | 49,919,254.67 ± 4,902,108.19 | 24,959,627.33 ± 2,451,054.10 | 74.34% ± 0.11% | 7,462,467,350.00 ± 730,282,325.17 | 46.96% ± 0.13% | 93.37% ± 0.22% |
| S4 | 51,239,034.67 ± 3,805,651.58 | 25,619,517.33 ± 1,902,825.79 | 74.65% ± 0.18% | 7,638,454,596.00 ± 532,291,639.10 | 46.40% ± 0.10% | 93.04% ± 0.17% |
| S5 | 44,837,081.33 ± 404,510.41 | 22,418,540.67 ± 202,255.20 | 74.65% ± 0.22% | 6,710,781,594.67 ± 60,785,954.35 | 47.50% ± 0.14% | 93.31% ± 0.15% |
| S6 | 47,676,506.00 ± 3,261,779.36 | 23,838,253.00 ± 1,630,889.68 | 74.95% ± 0.37% | 7,116,406,967.33 ± 466,746,406.33 | 47.18% ± 0.44% | 93.64% ± 0.28% |
| S7 | 47,836,408.67 ± 829,906.73 | 23,918,204.33 ± 414,953.36 | 75.27% ± 0.28% | 7,156,467,693.33 ± 126,560,192.05 | 48.00% ± 0.15% | 93.59% ± 0.20% |

Clean bases, total number of clean nucleotides; The GC% is the proportion of guanidine and cytosine nucleotides among total nucleotides; The Q30% is the proportion of nucleotides with a quality value > 20 and 30, respectively. S1, Control Shangshu-9; H1, Drought-treated Shangshu-9; S2, Control Chaoshu-1; H2, Drought-treated Chaoshu-1; S3, Control Xushu-22; H3, Drought-treated Xushu-22; S4, Control Z15-1; H4, Drought-treated Z15-1; S5, Control Xushu-18; H5, Drought-treated Xushu-18; S6, Control Jishu-26; H6, Drought-treated Jishu-26; S7, Control Xuzi-8; H7, Drought-treated Xuzi-8.

Table S2 All Database annotation of different sweet potato cultivars under drought condition.

Table S3 Overview of upregulated or downregulated genes in different sweetpotato genotypes under normal and drought conditions at a level of |log2 (fold change)| > 1 and FDR < 0.05.

| Sweet potato | Genes |  | Number of DEG | | | Number of annotated unigenes | | | | |
| --- | --- | --- | --- | --- | --- | --- | --- | --- | --- | --- |
|  | Genes | New genes | Total DEGs | up-regulated | down-regulated | Total DEG | COG | GO | KEGG | KOG |
| H1/S1 | 37851 | 7660 | 382 | 71 | 311 | 361 (94.50%) | 118 | 215 | 124 | 156 |
| H2/S2 | 37744 | 7550 | 1357 | 437 | 920 | 1291 (95.13%) | 493 | 856 | 539 | 645 |
| H3/S3 | 38515 | 7966 | 467 | 220 | 247 | 418 (89.51%) | 119 | 254 | 168 | 214 |
| H4/S4 | 41084 | 9268 | 1503 | 519 | 984 | 1431 (95.21%) | 572 | 958 | 564 | 756 |
| H5/S5 | 37745 | 7607 | 579 | 195 | 384 | 551 (95.16%) | 207 | 355 | 212 | 285 |
| H6/S6 | 40637 | 9131 | 674 | 420 | 254 | 644 (95.55%) | 268 | 372 | 237 | 327 |
| H7/S7 | 37499 | 7583 | 162 | 104 | 58 | 155 (95.68%) | 46 | 93 | 50 | 76 |

DEGs, Differentially expressed genes. H1/S1, drought-treated Shangshu-9/control Shangshu-9; H2/S2, drought-treated Chaoshu-1/control Chaoshu-1; H3/S3, drought-treated Xushu-22/control Xushu-22; H4/S4, drought-treated Z15-1/control Z15-1; H5/S5, drought-treated Xushu-18/control Xushu-18; H6/S6, drought-treated Jishu-26/control Jishu-26; H7/S7, drought-treated Xuzi-8/control Xuzi-8.

Table S4 The result of Venn.

Table S5 The result of KEGG enrichment in different sweet potato cultivars under drought condition
